# Gestion sécurisée des dépouilles de personnes décédées de la COVID-19 en Afrique sub-Saharienne: et si on laissait les familles enterrer leurs morts?

**DOI:** 10.11604/pamj.supp.2020.35.148.25253

**Published:** 2020-08-17

**Authors:** Moise Timtchueng, Clarisse Mapa-Tassou, Patrick Juvet Lowe Gnintedem, Hervé Martial Tchabo Sontang, Mireille Ndoungue, Vivien Meli, Henri René Zambou, Siméon Pierre Choukem

**Affiliations:** 1 Département de Droit Privé et Sciences Criminelles, Faculté des Sciences Juridiques et Politiques, Université de Dschang, Dschang, Cameroun,; 2 Health of Populations in Transition (HoPiT) Research group, Université de Yaoundé 1, Yaoundé, Cameroun,; 3 Département de Psychologie, UFR Sciences Humaines, Sociales et Philosophie, Université de Picardie Jules Verne, Amiens, France,; 4 Département de Philosophie, Psychologie et Sociologie, Faculté des Lettres et Sciences Humaines, Université de Dschang, Dschang, Cameroun,; 5 Equavet Group, Douala, Cameroun,; 6 Département de Médecine Interne et Spécialités, Faculté de Médecine et des Sciences Pharmaceutiques, Université de Dschang, Dschang, Cameroun,; 7 Service de Médecine Interne et Spécialités, Hôpital Général de Douala, Douala, Cameroun,; 8 Health and Human Development (2HD) Research Network, Douala, Cameroun

**Keywords:** COVID-19, inhumation, gestion des corps, législation, Afrique sub-Saharienne, Cameroun, COVID-19, burial, corpse management, legislation, sub-Saharan Africa, Cameroon

## Abstract

La pandémie à Coronavirus 2019 (COVID-19) touche les pays d´Afrique sub-Saharienne depuis le mois de mars 2020. Au-delà des désastres sanitaire et économique causés, se pose un problème psycho-socio-culturel en rapport avec la gestion des corps de personnes décédées de cette maladie; ce problème est susceptible d´entraver la bonne marche de la stratégie de riposte. Au Cameroun par exemple, la gestion actuelle de ces dépouilles ne fait pas l´unanimité. En effet, les restrictions appliquées à l´inhumation, bien que récemment assouplies proscrivent entre autres tout transfert interurbain des dépouilles. A la lumière des considérations culturelles africaines de la personne décédée, des dissensions créées entre les familles et le corps médical, de la législation et des données scientifiques disponibles, cet article analyse les risques et les bénéfices de l´inhumation des dépouilles par les familles. Il propose ensuite des solutions qui concilient la dignité (en laissant les familles enterrer leurs morts dans les domiciles), et la sécurité (en assurant une conservation hermétique et la surveillance d´un officier de police judiciaire). L´application de ces solutions pourraient améliorer la confiance de la population envers le système de santé et contribuer positivement aux stratégies de prévention, d´identification et de prise en charge des cas de COVID-19.

## Abstract (en)

Sub-Saharan African countries have been hit by the Coronavirus 2019 pandemic (COVID-19) since March 2020. Besides the resulting health and economic disasters is the psycho-socio-cultural problem related with the management of corpses of people dead from the disease, which might hinder the implementation of the response strategy. In Cameroon for instance, the current corpse management policy is very disputed. In fact, although they were recently made more flexible, the restrictions applied to burials still ban any transfer of dead bodies between cities. In light of the African cultural considerations of dead persons, the disputes observed between the families and the health personnel, the legislation and the available scientific evidence, this article analyses the risks and benefits of allowing families to bury their relatives. It thereafter suggests solutions that reconcile dignity (by allowing families to bury their dead relatives in their homes) and safety (by ensuring a sealed handling and the surveillance by a judiciary police officer). Applying these solutions could improve the population's trust towards the health system, and positively contribute to COVID-19 case prevention, identification and management.

## Essay

La pandémie de la maladie à Coronavirus 2019 (COVID-19) représente depuis plusieurs mois maintenant, un problème majeur de santé auquel est confrontée l'humanité toute entière non seulement en raison du nombre de personnes qui tombent malades et de la catastrophe économique qui s'ensuit, mais aussi en raison du grand nombre de décès. La situation mondiale au 25 juillet 2020 indique un total de plus de 16 millions de cas confirmés depuis le premier cas identifié en Chine en fin novembre 2019, et un nombre de décès qui dépasse déjà 640 000 [1]. Devant les ravages occasionnés par cette pandémie, des mesures de riposte en vue de limiter sa propagation ont été prises dans quasiment tous les pays du monde. L´une des caractéristiques communes à ces mesures a été de limiter considérablement les droits et libertés collectives et individuelles. Et dans l´optique de contraindre la population à s´y conformer, des mesures spéciales à caractère pénal ont dû être prises pour prévoir et réprimer le non-respect du confinement, à l´exemple de la France [2].

Au Cameroun par exemple, par une Déclaration spéciale du 17 mars 2020 [3], le Premier ministre rendait publiques les treize mesures de la stratégie gouvernementale camerounaise de riposte. Seulement, alors que la gestion des dépouilles des personnes décédées de la COVID-19 n´était énoncée nulle part dans le document publié après ladite déclaration spéciale, on va observer une confiscation desdites dépouilles aux familles pour être inhumées dans des cimetières publics sans l´accomplissement d´aucun rite funéraire usuel [4]. C´est le 23 avril 2020 que, par un autre communiqué, le Premier Ministre a instruit le ministre de l´Administration Territoriale, en relation avec le ministre de la Santé Publique, «*de s´assurer que les personnes décédées des suites du coronavirus soient inhumées dans leurs localités de décès*» [5]. L´interprétation littérale de ce communiqué commandait simplement que l´on ne permette aucun transfert interurbain des dépouilles et que la mise en terre se fasse sans délai, contrairement à la coutume de conserver les corps à la morgue pendant un ou plusieurs jours. Cependant, tout ceci a généré des problèmes allant du fait de cacher les malades (de peur de ne pas disposer du cadavre en cas de décès), à la violence contre le personnel de santé et aux tentatives d´exhumer les corps, tout ceci par les familles [6]. A la lumière des connaissances actuellement disponibles, et en prenant l´exemple du Cameroun, cet article fait une analyse des risques et bénéfices de laisser les familles enterrer leurs proches, et suggère des solutions pour assurer une gestion des corps qui concilie sécurité pour la santé publique, et dignité pour les familles et leurs coutumes.

**Rappel sur le statut juridique coutumier des morts en Afrique:** le statut du cadavre diffère selon que l´on se situe sous le prisme du droit civil hérité de la France ou sous une optique de droit coutumier. En France, on considère que «*le cadavre n´est pas une personne, ni en survie, ni en continuation de sa propre personne de défunt*» [7]. Pour la doctrine majoritaire, la dépouille mortelle est clairement une chose [8], même si on lui reconnait, pour un temps, les attributs d´une personne [9]. La chosification de la dépouille mortelle a pour conséquence d´en faire un objet de peu d´égards, même s´il mérite respect [10]. Quoique la catégorisation du cadavre en chose ne fasse pas l´unanimité [8], elle se fonde notamment sur l´idée qu´en droit civil, la personnalité juridique qui confère aux êtres humains leur qualité de sujet de droit commence à la naissance et s´achève avec la mort, faisant passer le défunt du statut de personne à celui de chose.

En Afrique et au Cameroun, la mort n´est pas vue comme la cessation de la vie, mais comme une simple mutation, un changement de monde. La cosmogonie africaine conçoit le monde sous une double dimension: le monde visible, celui des vivants; et le monde invisible, domaine des morts et des entités célestes [11]; la mort ici n´existe pas. C´est dans cet esprit qu´il faut comprendre le propos de Roger Kuipou lorsqu´il écrit qu´«*au moment du décès, on dit que le père s´est assoupi ou bien, qu´il s´est endormi. Pendant la période de deuil et jusqu´aux funérailles, on parlera de lui comme de celui qui dort là-bas ou bien celui qui est couché là-bas*» [12]. La métaphysique négro-africaine, bâtie sur le culte de la vie, de la force et de la richesse ontologique [13], mise au service de la création du droit milite en faveur de l´élaboration d´un statut juridique spécifique au cadavre en tant qu´il demeure un homme [14]. La catégorie de «*personne défunte*» ou de «*personne décédée*» proposée par d´habiles juristes français [8] semble mieux adaptée aux morts en Afrique. Demeurant des personnes [15], les morts mériteraient donc de bénéficier des égards comparables à ceux que l´on accorde à ceux qui sont encore en vie, au moins jusqu´à la désignation de l´héritier coutumier, continuateur de la personne du de cujus [16]. Dans cet esprit, les dépouilles mortelles devraient au minimum être traitées comme des personnes en état d´incapacité qui, à défaut d´exprimer elles-mêmes une volonté efficace, agissent par l´entremise d´un représentant choisi parmi les membres de la famille.

**Législation sur l´inhumation et nécessité de procédures opératoires standards de gestion des corps en contexte d´épidémie: cas du Cameroun:** au Cameroun, légalement seul le "décret 74/199 du 14 mars 1974 portant réglementation des opérations d´inhumation, d´exhumation et de transfert de corps" définit clairement comment gérer les corps de personnes décédées de maladie contagieuse, et même le délai d´exhumation en cas de nécessité. Or, ni les communiqués, ni les recommandations, ni les avis scientifiques qui ont guidé à ce jour la gestion des corps dans le cadre de l´épidémie à COVID-19 ne peuvent remplacer ledit décret. Leur but devrait être de compléter ou renforcer ce décret, tout en restant en conformité avec lui. L´épidémie de la COVID-19 semble pouvoir persister pendant plusieurs mois encore, voire années; les changements environnementaux et la fréquence des précédentes épidémies au cours des dernières décennies laissent tous penser qu´il y aura davantage d´épidémies zoonotiques. Tout ceci plaide en faveur de l´élaboration par des équipes multidisciplinaires, de procédures opératoires standards (POS) pour la gestion des corps de personnes décédées suites auxdites maladies zoonotiques. Ces POS devraient clairement, entre autres: 1) Définir les rôles et responsabilités de chaque décideur dans le processus du strict respect des obligations sanitaires et socio-culturelles; 2) Décrire les procédures de manipulation avec les responsabilités du personnel de santé, de l´administration, des autorités judiciaires et des forces de l'ordre, et des familles; 3) Valider les équipements de protection, les désinfectants et procédures de désinfection, les moyens et procédures de conservation, le mode et les procédures de transport, et les conditions éventuelles d´exposition, d´hommage et d´inhumation.

**Impopularité de la mesure prescrivant l´inhumation immédiate:** le deuil de la perte d'un être cher pendant cette période stressante de la pandémie de la COVID-19 peut être accablant. Le deuil est une réponse normale à la perte d'une personne importante pour soi. Lorsqu'un proche décède, il est important que les amis et la famille puissent partager des histoires et des souvenirs de la personne. En raison de la pandémie de la COVID-19, la distanciation sociale, les recommandations de « Restez chez vous » et les limites des rassemblements ont affecté la capacité des amis et de la famille à se réunir et à pleurer de manière typique [17]. Au Cameroun, la mesure prescrivant l´inhumation immédiate des dépouilles victimes de la COVID-19 passe mal dans l´opinion publique; les familles ne s´y soumettent que très péniblement. Certaines n´hésitent pas à recourir à la violence pour essayer de récupérer à tout prix la dépouille du leur, aux fins de l´inhumer elles-mêmes comme d´usage [18]. La confiscation des dépouilles est vécue comme « un drame psychologique redoutable », dans la mesure où «*chez les Africains, la famille a besoin de faire son deuil avec le corps du défunt. De ce fait, lorsque les dépouilles ne sont pas remises aux familles, les gens auront du mal à faire leur deuil, surtout si ces gens ne savent pas comment des dépouilles ont été inhumées*» [19].

Même si la mesure est/était également pratiquée ailleurs [20], les divergences de considérations données aux morts, de même que les différences au niveau des systèmes de santé en Europe et en Afrique, n´encouragent pas à appliquer, partout, des solutions identiques. De plus, les positions ont progressé dans de nombreux pays. En Algérie par exemple, depuis fin mai 2020, le gouvernement a fixé les modalités de rapatriement des dépouilles des Algériens décédés du coronavirus à l´étranger, à travers un arrêté interministériel [21]. Les solutions doivent donc constamment être adaptées en fonction des milieux concernés, de façon à s´assurer «*l'adhésion et la collaboration des citoyens au plan individuel et collectif pour une meilleure efficacité*» [22]. Si la nécessité des mesures barrières (lavage régulier des mains ou utilisation du gel hydroalcoolique, port de masque, distanciation sociale, etc.) et de la fermeture des frontières peuvent universellement se comprendre, le principe de l´inhumation des personnes victimes de la COVID-19 mérite d´être contextualisé pour tenir compte de la considération accordée aux morts dans l´environnement dans lequel on se trouve. L´on s´accorde à reconnaître que la famille constitue un secteur de «*faible perméabilité*» [23] à l´emprunt des règles élaborées ailleurs, parce qu´elle constitue «*le domaine par excellence des mœurs traditionnelles, de la coutume et de la religion*» [24]. La dépouille mortelle au centre de la présente réflexion est/était membre d´une famille. Alors, il importe que les règles mises en œuvre pour son traitement tiennent compte de la place qu´elle occupe dans les mœurs et en droit coutumier.

### Balance risque/bénéfice de l´inhumation des corps de la COVID-19 suivant les coutumes

***Risque «COVID-19» lié à la gestion des corps:*** en l´état actuelle des connaissances, le Coronavirus responsable de la COVID-19 pénètre dans l'organisme par les muqueuses buccales, nasales ou oculaires et infecte le système respiratoire. La transmission se fait par les sécrétions lors de la toux, des éternuements ou de la parole [25], et le nombre de reproduction de base de COVID-19 est de 2,5 à 3 [26]. Autrement dit, toute personne infectée infecterait deux à trois personnes. Nous avons retrouvé dans la littérature un seul cas rapporté de contamination d'un médecin légiste en Thaïlande par un cadavre qui a probablement été mal manipulé, ce qui rappelle l´obligation à porter un équipement de protection individuelle approprié [27]. Selon l´Organisation Mondiale de la Santé (OMS), « seuls les poumons peuvent être contagieux s´ils ne sont pas manipulés correctement au cours d´une autopsie. Sinon, les cadavres ne transmettent pas la maladie ». Bien que le risque de contamination par une personne décédée est faible, de nombreuses personnes ont été contaminées par le coronavirus après avoir assisté à un service funéraire dans certains pays [28]. Pour aider à prévenir la propagation du COVID-19 dans les communautés, des changements doivent être apportés à la façon dont les funérailles sont organisées.

En fait, le vrai risque de contamination par la COVID-19 posé par les inhumations semble de toute évidence être celui liée aux rassemblements que ces inhumations (quelle que soit la cause du décès) induisent, plutôt qu´au risque lié au cadavre. Dans cette optique, ce sont les rassemblements qui doivent être mis en avant, ce d'autant les décès liés aux causes autres que la COVID-19 continuent et sont gérés par les familles comme d´habitude, souvent au mépris des mesures de distanciation. Dans cette optique, encourager la conservation prolongée des corps dans les morgues quelles que soient la cause, pour les familles qui le souhaitent, pourrait d´ailleurs être l´une des solutions efficaces, parce que d´une part elle contribuerait à diminuer les rassemblements, et d´autre part elle permettrait aux familles de rendre sans stress le type d´hommage souhaité au leur qui est décédé, à distance du pic de l´épidémie. Une autre raison qui aurait pu sous-tendre les restrictions drastiques en matière d´inhumation au cours d´une épidémie, c´est la survenue de décès massifs qui seraient très complexes à gérer si on laissait les familles inhumer leurs morts. Mais dans le cas de l´actuelle crise sanitaire, le Cameroun par exemple a enregistré 385 décès au 25 juillet 2020, soit une moyenne de 19 décès par semaine attribuable à la COVID-19 depuis le diagnostic du premier cas local. A titre de rappel, en 2018 la moyenne hebdomadaire du nombre de décès total au Cameroun, calculée à partir des données de mortalité, était d´environ 4495 [29]. Avec un recul de plus 4 mois, on peut donc dire que le risque de décès massifs observés dans certains pays occidentaux n´a pas lieu, et ceci est vrai dans la plupart, sinon tous les pays d´Afrique sub-Saharienne. Et au demeurant, si une telle situation survenait dans le futur, il suffirait de réajuster et adapter la stratégie.

***Risque de traumatisme lié au vécu des familles suite à la gestion actuelle des corps COVID-19:*** le processus de deuil et les rituels formels et informels connexes, à travers lesquels les personnes endeuillées pleurent le décès de leurs proches, sont importants pour la santé et le bien-être des personnes endeuillées. Les familles et les proches vivent mal la manière dont les corps des personnes décédées de la COVID-19 sont enterrés jusqu´ici [18]. Comme nous l´avons présenté plus haut, au Cameroun, les familles sont tenues à rendre un hommage digne aux défunts et les dérogations à se devoir peuvent avoir un impact personnel et social. Dans cette logique, on ne devrait jamais exclure la famille du processus d´inhumation d´une personne décédée de la COVID-19. Aussi longtemps que la dépouille reste une personne, préséance doit revenir à sa famille chaque fois que du fait de la mort, elle se trouve en situation d´incapacité juridique.

**A la recherche des solutions conciliantes:** de nombreuses organisations ont recommandé d'appliquer les techniques d'enterrement habituelles, qui permettent de laisser la famille suivre un minimum de choix culturels spécifiques. L'OMS, par exemple recommande, sur la base des preuves disponibles en particulier des précédentes épidémies de grippe, de permettre à la famille d'appliquer les principes de sensibilité culturelle et de suivre leurs coutumes, à condition que toutes les mesures d'hygiène et de distanciation sociale soient appliquées avant, pendant et après l'enterrement [28]. Elle met l´accent sur la dignité des défunts et leurs familles, qui doit être respectée et protégée tout au long du processus de prise en charge [28]. Le *Center for Disease Control and Prevention*(CDC) va dans le même sens de respect des mesures barrières, en demandant que les obsèques des défunts de COVID-19 soient organisés par la famille sur haute surveillance des personnes habilitées à manipuler les corps, et que les traditions et coutumes des défunts et de leurs familles soient respectées [17].

Au Cameroun, de nombreuses voies dans le monde médical et scientifique se sont levées pour appeler à une inhumation décente par les familles. L´Académie des Sciences du Cameroun dans sa deuxième déclaration sur la réponse du Cameroun à la pandémie de la COVID-19 relève en son point 15 «*Demande au Gouvernement d'envisager de suivre les directives de l'OMS sur la gestion sûre du cadavre dans le cadre de COVID-19; ces directives, en effet, permettent un enterrement sûr et sécurisé (avec des cercueils scellés au zinc) par la famille selon ses coutumes. Ce point est très sensible car il est à l'origine de la violence généralisée et croissante à l'encontre du personnel de santé*». Devant les incompréhensions, réclamations et déviances liées à la gestion des corps de personnes décédées de la COVID-19, le ministre de la santé Publique du Cameroun, en date du 12 mai 2020, a saisi le conseil scientifique des urgences sanitaires sur la question. Ce conseil s´étant appuyé sur les orientations provisoires de l´OMS, a recommandé que le défunt pouvait alors être inhumé dans un délai de 48 heures, « dans le respect de la dignité humaine et de leurs traditions culturelles et religieuses », dans le domicile familial s´il en a dans la ville dans laquelle est survenu le décès, en présence de quelques membres de la famille et proches dans le respect des mesures de distanciation et surtout que les corps soient placés «dans un cercueil hermétique, fermé et zingué, avec ou sans vitre encastrée permettant à la famille de voir le visage du défunt», et enterrés dans le respect de la dignité [30].

Ces recommandations du conseil scientifique des urgences sanitaires apportent un assouplissement dans les procédures d´inhumation des dépouilles. Néanmoins les familles restent dévastées par l´incapacité d´inhumer leurs défunts dans leurs villages. Face à cette situation désolante, d´autres mesures d´assouplissement s´avèrent fortement recommandées pour une inhumation des cadavres des personnes COVID-19, qui concilie une totale sécurité sur le plan de la santé publique, et une totale dignité pour les familles et leurs coutumes. Les étapes suivantes pourraient être suivies: 1) Permettre la conservation du corps à la morgue pendant toute la durée souhaitée par la famille; 2) Sceller et zinguer le cercueil; 3) Autoriser le voyage si nécessaire, sous la surveillance d´un officier de police judiciaire aux frais de la famille; 4) Prohiber l´autopsie traditionnelle et toute autre forme de manipulation du corps par la famille; 5) Accorder 24 heures de séjour dans la famille (dans la localité où le corps le corps va être inhumé); 6) Laisser inhumer dans le domicile familial dans le strict respect des mesures-barrières, le tout sous la surveillance d´un officier de police judiciaire, aux frais de la famille.

Pour les personnes déjà inhumées dans les conditions indécentes, appliquer le décret 74/199 du 14 mars 1974 en accordant la possibilité d´exhumation après un minimum de 3 années posthumes pour poursuivre les cérémonies culturelles. Ces propositions émanent d´une réflexion holistique impliquant juristes, médecin, psychologue, sociologue, spécialiste des zoonoses, et expert de santé publique et politiques de santé; elles visent à équilibrer les besoins des personnes endeuillées à pleurer de manière appropriée, tout en minimisant la propagation de l'infection par le coronavirus. Par ailleurs, un plan de communication clair intégrant tous les acteurs, aussi bien communautaires, traditionnels, religieux, scientifiques que du secteur médical, doit être mis en place pour faciliter la compréhension et l´acceptation de ces propositions. Pour mieux soutenir ces propositions, la production des données locales sur l´impact psychologique de la situation actuelle de la gestion des dépouilles au Cameroun et dans d´autres pays d´Afrique sub-Saharienne est un impérative. De plus, la mise en place de ces propositions doit s´accompagner d´une étude risque/bénéfice de l´inhumation des dépouilles de la COVID-19 suivant les coutumes africaines. Ces données sont importantes tant pour la gestion de l´épidémie actuelle que pour les épidémies futures.

## Conclusion

La gestion actuelle très restrictive des corps de personnes décédées de la COVID-19 heurte la dignité culturelle africaine, crée des tensions entre d´un côté les familles et de l´autre le corps médical et les autorités, et n´est par ailleurs justifiée par aucune base scientifique ni légale. Dans le strict respect des mesures barrières, des dispositions peuvent être prises pour le transfert sécurisé des dépouilles vers les villages, associé à la prohibition de toute forme de manipulation du corps par des tiers. Ces propositions permettent de concilier sécurité et dignité; leur application augmenterait la confiance des populations envers le système de santé et contribuerait positivement à la lutte contre la pandémie.
